# Herniation of the Right Colon, From the Cecum to the Hepatic Flexure, Through the Foramen of Winslow: A Case Report

**DOI:** 10.7759/cureus.61387

**Published:** 2024-05-30

**Authors:** Austin M Lundgren, Jordan P Fader, Griffin A Dufek, Ani G NaThalang-Piñon

**Affiliations:** 1 Surgery, A.T. Still University, Kirksville, USA; 2 Surgery, Kansas City University, Kansas City, USA; 3 General Surgery, University of Missouri Capital Region Medical Center, Jefferson City, USA

**Keywords:** case report, cecum, right colon, bowel obstruction, internal hernia, foramen of winslow

## Abstract

Herniation of bowel contents between the peritoneal cavity proper and the omental bursa, through the foramen of Winslow, can present diagnostic challenges that can potentially delay necessary surgical intervention. This case describes a 49-year-old female with a past medical history of hiatal hernia and biliary dyskinesia who presented to the emergency department with severe epigastric and right lower quadrant abdominal pain one day after a reported gastrointestinal illness of unknown etiology. Initial emergency department workup demonstrated an elevated white blood cell count without lactic acidosis. Computed tomography imaging was interpreted as gastric distension with volvulus around the mesentery and second portion of the duodenum. Intraoperatively, the entirety of the right colon was noted to have passed through the foramen of Winslow into the lesser sac. This led to twisting of the mesocolon causing compression of the duodenum and a gastric outlet obstruction. After surgical reduction of the herniation, the patient noted great improvement in pain and other symptoms.

## Introduction

The foramen of Winslow (FoW) is an anatomical opening connecting the greater and lesser peritoneal cavities [1]. The boundaries of the foramen are the free edge of the lesser omentum anteriorly, the inferior vena cava posteriorly, the caudate lobe of the liver superiorly, and the duodenum inferiorly [1]. Hernias through the FoW account for 0.08% of all hernias and 8% of internal hernias, which are hernias occurring within the peritoneal cavity [1,2]. The peak incidence of FoW hernias is between the third and sixth decades of life [2]. More than 60% of hernias through the FoW contain only small bowel [1]. The remainder contains some combination of the small bowel, cecum, ascending colon, transverse colon, omentum, and gallbladder [1]. Treatment is surgical with both open abdominal and laparoscopic techniques [2]. There is still debate about whether prophylactic FoW closure or fixation of the herniated contents should be routinely performed [2].

## Case presentation

We describe the case of a 49-year-old female who presented to the regional emergency department (ED) with severe epigastric and right lower quadrant abdominal pain. The patient reported a past medical history of hiatal hernia and biliary dyskinesia. History obtained in the ED noted pain beginning one day after a reported gastrointestinal illness of unknown etiology that led to nausea, more than 20 episodes of vomiting without hematemesis, and diarrhea. On the day of hospital admission, the patient had passed flatus but no bowel movements. She presented with an elevated blood pressure at 208/110 mmHg and a respiratory rate of 38 breaths per minute. She did not have fever and chills, and the rest of her vital signs were within normal limits.

The ED workup included laboratory analysis and imaging. Laboratory analysis revealed a white blood cell count of 16,000 per microliter (normal range: 4,500-11,000 per microliter) [3] and lactic acid less than 1 mmol/L (normal range: less than 2 mmol/L) [4]. Additionally, serum potassium was noted to be 2.8 mmol/L (normal range: 3.5-5.0 mmol/L) [5], and the glomerular filtration rate (GFR) was 26 mL/min (normal range: greater than 90 mL/min) [6]. Blood pressure and respiratory rate slightly decreased during her time in the ED from initial readings to 179/107 mmHg and 20 breaths per minute, respectively. The patient still appeared to be in pain, but the rest of the physical exam showed no signs of an acute abdomen. The radiology report from a computed tomography (CT) scan without contrast demonstrated gastric distension with volvulus around the mesentery and second portion of the duodenum. General surgery was then consulted and advised the ED to trial nasogastric (NG) tube placement. On chest X-ray, the tip of the NG tube was visualized at the gastroesophageal junction. The general surgeon tried to advance the NG tube before encountering resistance and stopping, without any return of gastric contents in the NG tube.

**Figure 1:**
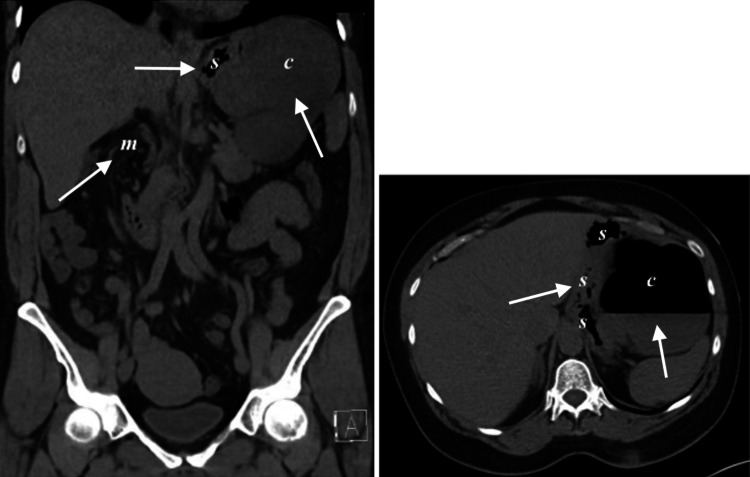
Coronal and axial preoperative CT scans with labels of anatomy discovered during the hernia reduction CT: computed tomography; m: mesocolon twisting, c: cecum and right colon, s: stomach

The decision was made to proceed immediately to surgery. A robot-assisted laparoscopic approach was taken. Upon abdominal entry, the stomach was very dilated, limiting visualization. Needle decompression of the stomach was performed followed by suture closure with 2-0 Vicryl absorbable suture after an unsuccessful attempt to pass an NG tube intraoperatively. Then, an esophagogastroduodenoscopy (EGD) was performed, and the scope was advanced to the third portion of the duodenum without evidence of twisting. After ensuring there was no evidence of volvulus, contents were still noted to be within the lesser sac. These contents were discovered to be the entire right colon from the cecum to the hepatic flexure (see Figure 1 for the labeled CT scan with anatomy labeled as visualized during surgery). The lesser sac was opened, and the herniated colon was visualized. After performing needle decompression of the colon, it was passed through the FoW and pexied to the paracolic gutter using a 2-0 Vicryl absorbable suture, ensuring that there were no signs of necrosis and that the colon segment was in proper anatomical position. While a common treatment option is to perform a right hemicolectomy, the decision was made to keep the entire colon intact as the patient was relatively healthy, there was no sign of necrosis, and time was needed to determine if the herniation was the only cause of the patient's pain. As the initial imaging indicated a possible gastric volvulus, the stomach was pexied to the anterior abdominal wall using a 2-0 Vicryl absorbable suture in an effort to reduce the chance of future volvulus.

The patient tolerated the procedure well. Due to the hypokalemia diagnosed in the ED, she was given 40 mEq of intravenous potassium. The following day, her potassium increased to 4.3 mmol/L (normal range: 3.5-5.0 mmol/L) [5]. When rechecked, her GFR improved to greater than 60 mL/min, the highest value obtainable in the electronic health record.

After spending a few days in the hospital, the patient was discharged home with minimal postoperative discomfort. At the initial outpatient follow-up two weeks post-discharge, she was recovering well with no residual symptoms. She will continue to have regular follow-ups with general surgery and colonoscopy planned for three months post-surgery to monitor for bowel changes or obstruction after the hernia reduction.

## Discussion

From a physiological standpoint, the initial hypokalemia and hypertension resolved during hospitalization. The patient was hypokalemic, with a serum potassium level of 2.8 mmol/L upon arrival to the ED. Hypokalemia is common after vomiting, as vomiting induces hypochloremic metabolic alkalosis due to the loss of hydrochloric acid from gastric contents [7]. In cases of severe vomiting and nasogastric suctioning, volume depletion contributes to hypokalemia by activating the renin-angiotensin-aldosterone system, which enhances renal potassium wasting [8]. Additionally, it has been established that acute pain leads to increased systolic and diastolic blood pressures [9]. This patient was in pain when she arrived to the ED where she was found to be hypertensive. As pain resolved with treatment, the blood pressure decreased to within normal limits. This demonstrates that patients with acute pain do not always necessitate medical hypertension management.

From a surgical standpoint, this is a rare case of right colon herniation from the cecum to the hepatic flexure through the FoW into the lesser sac. The small bowel is the most common hollow viscus involved in FoW hernia, accounting for more than 60% of cases [1,10]. The cecum and right colon are the next most commonly involved at approximately 30% of cases, and the transverse colon is the rarest at 7% of cases [10]. Hernias through the FoW are seldom diagnosed preoperatively, but techniques for identifying them on CT have been described [1,10].

The initial reading of the CT scan indicated gastric distension with volvulus around the mesentery and second part of the duodenum; however, surgical exploration did not yield signs of gastric volvulus and instead revealed colonic herniation through the FoW. In the literature, CT findings associated with hernias through the FoW include air-fluid levels in the lesser sac [1,11], anterior and lateral displacement of the stomach [1,11], stretching of mesenteric vessels through the foramen [1,11], two or more bowel loops in the high subhepatic space [11], absence of cecum and ascending colon in the right gutter [11], and herniated cecum having the appearance of a cecal volvulus [1].

The importance of accurate diagnosis in this case is paramount, as the initial and final diagnoses are treated differently. Standard treatment for gastric volvulus involves NG tube placement [2,12]. When the right colon herniates through the FoW, prompt surgical exploration and hernia reduction are necessary; however, there does not appear to be a general consensus on whether fixation of the colon to the lateral abdominal wall or right hemicolectomy is preferred [13-15]. As was performed in this case, fixation of the colon to the lateral abdominal wall with suture has been proven safe [13]. Some argue that performing a right hemicolectomy is superior, as it both excludes underlying intraluminal pathology and prevents the recurrence of hernia by removing the lengthened mesentery [15].

It is important to consider biases that impact medical decision-making. Anchoring bias, failing to adjust an initial impression despite receiving additional information, and premature closure, accepting an initial diagnosis as final, are examples of biases that contribute to radiologic misinterpretation [16]. Bias can also occur after a diagnosis has been confirmed, as hindsight bias is the tendency to retrospectively de-emphasize the difficulty in making the initial diagnosis after its confirmation [16]. Being aware of potential biases can improve metacognition and reduce cognitive diagnostic errors. 

## Conclusions

Heightened awareness of the clinical symptoms and radiologic findings of FoW hernias can contribute to prompt identification and treatment. While there is not a consensus on which surgical technique is superior, it is evident that prompt surgical intervention and reduction of the hernia are necessary.

This case exemplifies how clinicians must synthesize all of the information presented when making treatment decisions. While initial imaging interpretation could have pushed treatment down one path, considering the lack of response with NG decompression and reviewing images independently raised enough clinical suspicion to proceed to surgery. As clinicians work to provide high-quality care to their patients, it is important to realize that even with years of experience, findings can be misinterpreted or overlooked. Having a high index of clinical suspicion and questioning factors that do not fit the whole picture are imperative for quality patient care.
